# Systems Biology in Fungal Research

**DOI:** 10.3390/jof8050478

**Published:** 2022-05-04

**Authors:** Jennifer Geddes-McAlister

**Affiliations:** Department of Molecular and Cellular Biology, University of Guelph, Guelph, ON N1G 2W1, Canada; jgeddesm@uoguelph.ca

**Keywords:** fungal pathogenesis, proteomics, genomics, transcriptomics, metabolomics, multi-OMICs, host, disease

## Abstract

The beauty within biological systems can be uncovered using a variety of advanced technological platforms for in-depth profiling. Improvements in genome, transcriptome, proteome, and metabolome investigations, as well as data integration, are moving our understanding of diverse biological systems forward at a rapid rate. Combined with publicly available and customizable bioinformatics tools, we comprehensively profile biological changes under a plethora of circumstances. For fungal pathogens innovation is driven by our ability to explore mechanisms of antifungal resistance, reveal new relationships and interactions between a host and pathogen, improve our characterization of virulence determinants, and discover new antifungal targets. In this Special Issue dedicated to “Systems biology in fungal research”, we explore each of these factors and more, highlighting the multitude of avenues and strategies available to study fungal pathogens and how they impact our environment.

The exploration of environmental sources (e.g., invertebrates, plants, microbes) for novel compounds with properties as biocontrol agents is a rapidly expanding area of study. For instance, biocontrol agents can impact disease management and progression, promotion of fungal growth, and mitigation of climate change. The discovery of enzymatic inhibitors from the environment with activity against fungal extracellular and intracellular proteases represents potential new antifungal options that may reduce virulence without supporting the evolution of resistance [1]. For a systems overview of proteases and their perspective inhibitors, mass spectrometry-based proteomics combined with enzymatic assays can detect and characterize the relationship between enzyme production and inhibition. Similarly, within the natural environment, a bacterium, *Lactiplantibacillus plantarum*, was evaluated for its antifungal activity against *Ascosphaera apis*, an entomopathogenic fungus causing chalkbrood disease in honeybees [2]. Beneficial antagonistic properties against *A. apis* were observed and the bacterium was proposed as a probiotic in honeybee nutrition to prevent the development of disease. Genomics and transcriptomics approaches can be used to select bacterial strains with beneficial properties, along with proteomics and metabolomics to assist with elucidating mechanisms of action for the perspective probiotics and provide new insight into the relationship between the host (i.e., honeybee), pathogen (i.e., *A. apis*), and treatment (i.e., *L. plantarum*).

Other opportunities to leverage a beneficial relationship between microbe and host includes assessment of bioprotective fungal endophytes within global crops. For example, the vertically transmitted fungal endophyte, *Epichloë* spp. produces alkaloids that protect grasses from herbivory and confer protection from drought and nutrient stress [3]. For perennial ryegrass (*Lolium perenne*), a cool-season grass with global distribution for its roles in carbon fixation, turfgrass applications, and fodder for livestock, the interaction with endophytes may protect the grass from rising atmospheric CO_2_ associated with climate change. In the study, integration of untargeted metabolomics profiling with bottom-up mass spectrometry-based proteomics, defined the endophyte-specific responses of *L. perenne* under normal (400 ppm) vs. elevated (800 ppm) CO_2_ levels that influenced plant biomass through the activation of general plant defense responses. A complementary study explored endophyte-strain-dependent chemical phenotypes in *L. perenne* using untargeted metabolomics [4]. Metabolome profiling of root exudates (with roles in nutrient acquisition, microbial associations, and below-ground defense) under the influence of four *Epichloë* spp. strains relative to an endophyte-free control defined chemical changes in both primary and secondary metabolites of the plant. Specifically, two compounds were each strongly associated with a specific-endophyte treatment, supporting changes in the chemical composition of root exudates, which may confer beneficial outcomes for the plant (e.g., improved nutrient uptake, enhanced defense responses).

Beyond grasses, the application of endophytes to prevent disease in fruit, including apple replant disease, is an important global area of study. Recently, an antagonistic endophytic fungus was collected from the roots of healthy apple trees and assessed for inhibition of fungal pathogens, such as *Fusarium* spp. [5]. The study identified *Trichoderma asperellum* 6S-2 as an effective biocontrol agent against disease in apples through production of protease, amylase, cellulase, and laccase, which are important for pathogen inhibition. Application of fermented extract from the 6S-2 endophyte demonstrated antifungal activity (e.g., the inhibition of *Fusarium* spp. hyphae formation) through the production of volatile substances, such as alkanes, alcohols, furanones, and 6-PP, which were identified by gas chromatography–mass spectrometry. Furthermore, the application of an endophytic spore suspension on replanted apple orchard soil within a growth pot reduced plant oxidative damage and promoted plant growth, supporting 6S-2 as an effective biocontrol against *Fusarium* spp. in apple disease. The same research group furthered our understanding of *T. asperellum* 6S-2 as a biocontrol agent by producing a fungal fertilizer and assessing global impact on the soil microbial community and seedling growth [6]. Following fertilizer application, the study observed a significant change in soil structure with a decrease in the abundance of environmental fungi (e.g., *Fusarium* spp., *Cryptococcus* spp.), compared to an increase in abundance of environmental bacteria (e.g., *Bacillus* spp., *Streptomyces* spp.). These community shifts also impacted soil nutrient availability, and fertilizer application led to increased amounts of alkenes, ethyl esters, and citrullines in the root exudates of apple seedlings, which positively correlated with the abundance of 6S-2. Taken together, these studies demonstrate the interconnectivity of the environmental sources of biocontrol agents and their potential for antifungal properties to promote the health of globally important biological systems, including honeybees, grasses, and fruit. The use of systems biology to identify and characterize these biocontrol agents, as well as profile their impact on the community, is critical to advance our understanding of biological mechanisms and promote safe and effective treatment options.

An important source of fungal pathogens causing disease within humans is from the environment, as is the case for *Cryptococcus* spp. Importantly, the exposure of environmental pathogens to fungicides in the field often influences the evolution of antifungal resistance, providing hurdles for the treatment of such infections within the clinic [7]. This relationship between the environment and clinic drives the need for new treatment strategies. For *Cryptococcus* spp. and other human fungal pathogens, such as *Candida* spp. and *Aspergillus* spp. the ability to cause disease is driven by a combination of virulence factors and manipulation of the host. Post-translational modifications (PTMs), which are well-defined using mass spectrometry-based proteomics, allow fungal pathogens to modulate protein production and activity during infection to respond to stress and promote fungal survival [8]. For example, the regulation of heat shock proteins by PTMs (e.g., acetylation) are important targets for antifungal drug design, given their roles in metabolic networks that regulate proper protein folding, growth inhibition, enzymatic activity, and thermotolerance [9]. Characterization of heat shock protein networks in human fungal pathogens is well underway for the detection of interactions within the pathogen, and between the pathogen and host, that regulate pathogenesis and tolerance to antifungals. This approach suggests a promising strategy for novel antifungal therapy through disruption of heat shock proteins.

Other strategies used by fungi to regulate the host include response and adaptation to changing environmental conditions. For instance, upon antibiotic treatment, which causes dysbiosis of the gastrointestinal tract, a disruption in the commensal relationship between the host and fungal pathogen, *Candida albicans*, can be observed. One effect is a significant increase in the production of a major bile acid (i.e., taurocholic acid,) which enhances susceptibility of mice to *C. albicans* infection by inducing colonization and dissemination of the fungi throughout the host [10]. The study also showed a change in the commensal bacterial population within the gastrointestinal tract of the host upon taurocholic acid treatment, which supports the regulatory role commensal bacteria play in promoting host homeostasis in the presence of fungal pathogens. Further exploration using multi-OMICs tools may provide new insight into how the exposure of fungi and bacteria to taurocholic acid promotes fungal adaptation and suggests opportunities to reverse such changes as a novel therapeutic option.

Considering such fungal adaptation strategies to changing environments and the increasing rates of antifungal resistance on a global scale, new methods to identify novel druggable targets with specificity for fungi are needed. Recently, a genome-scale metabolic model of *C. albicans* was developed and validated for the identification of druggable sites through the comparison between known drug targets and the prediction of gene essentiality in conditions mimicking the human host [11]. The modeling of metabolism and metabolic networks is a powerful strategy to build upon our fundamental knowledge of fungal pathogens and to explore the role of metabolite production within the biological system. For *Aspergillus niger*, altered metabolite production was observed upon regulation of a novel biosynthetic gene cluster associated with transcriptional regulation, which was identified following comparative genomics analyses [12]. The anchoring of the biosynthetic gene cluster by a nonribosomal peptide synthetase is notable due to bioactive roles (e.g., toxins, siderophores, pigments, antibiotic, cytostatics, immunosuppressants, or anticancer agents). Similarly, another biosynthetic gene cluster in *Aspergillus terreus*, encoding for monacolin J, was integrated into the *A. niger* genome to promote synthesis of the precursor of statin drugs (e.g., lovastatin, simvastatin) used to lower cholesterol [13]. This manipulation of the fungal system for optimal compound production supports advancement within industrial fermentation processes for food and medicine. Further engagement of such biological systems with systems-level profiling using multi-OMICs tools is a promising strategy to optimize and improve the engineering potential for diverse beneficial outcomes.

Overall, this Special Issue highlights the dynamic and diverse applications of OMICs tools, along with their potential, to advance our foundational understanding of fungal biology. Furthermore, such advancements provide great benefit to many facets of life, including our environment, food sources, medical therapies, and industrial production systems, encouraging the continued exploration and integration of knowledge generated from diverse datasets and biological systems.
